# Limitations of Current *in Vivo* Mouse Models for the Study of Chikungunya Virus Pathogenesis

**DOI:** 10.3390/medsci3030064

**Published:** 2015-08-07

**Authors:** Yi-Hao Chan, Fok-Moon Lum, Lisa F. P. Ng

**Affiliations:** 1 Department of Biochemistry, Yong Loo Lin School of Medicine, National University of Singapore, 21 Lower Kent Ridge Road, Singapore 117597, Singapore; E-Mails: yihao_chan@immunol.a-star.edu.sg (Y.-H.C.); Lum_Fok_Moon@immunol.a-star.edu.sg (F.-M.L.); 2 Laboratory of Microbial Immunity, Singapore Immunology Network, Agency for Science, Technology and Research (A*STAR), 8A Biomedical Grove, #04-06 Immunos, Singapore 138648, Singapore

**Keywords:** alphavirus, chikungunya, mouse models, pathogenesis, immunity

## Abstract

Chikungunya virus (CHIKV) is an arthropod-borne alphavirus that causes febrile chikungunya fever (CHIKF) in humans. This disease is debilitating and characterized by acute fever onset and chronic incapacitating polyarthralgia. CHIKF pathogenesis remains poorly defined with no approved vaccines and therapies. Recent outbreaks in the Caribbean islands have elevated concerns over the possibility of a global pandemic. Tremendous efforts have been made to develop relevant mouse models to enable the study of infection and immunity against this viral disease. Among them, the more common C57BL/6 mouse model demonstrated the ability to recapitulate the symptoms shown in infected humans, including self-limiting arthritis, myositis, and tenosynovitis. This has facilitated the unraveling of some key factors involved in disease pathogenesis of CHIKF. However, the stark differences in immune response between humans and mouse models necessitate the development of an animal model with an immune system that is more genetically similar to the human system for a better representation. In this paper, we aim to uncover the limitations of the C57BL/6 model and discuss alternative mouse models for CHIKV research.

## 1. Introduction

Chikungunya Virus (CHIKV) is an arthropod-borne alphavirus belonging to the *Togaviridae* family, and it was first isolated from an infected patient during an epidemic in Tanzania, East Africa in 1952 [1,2]. This enveloped virus contains a positive sense single-stranded RNA of 11.8 kb, and can be efficiently maintained in both the sylvatic or urban cycles [3,4]. Within the urban cycle, CHIKV is primarily transmitted from person-to-person through the *Aedes aegypti* or *albopictus* mosquitoes [5,6,7,8]. CHIKV infection is usually non-fatal and self-limiting, and can be categorized into two phases: acute phase and chronic phase [4]. During the acute phase, CHIKV-infected patients exhibit hallmark clinical features such as fever, headache, rash and arthralgia 2 to 7 days post-infection [3,9]. However, approximately 30% of infected individuals continue to experience prolonged symptoms during the chronic phase, which may persist for years [4]. Chronic manifestations include fatigue, chronic arthritis, myositis, and tenosynovitis [4,10,11]. The pathogenesis remains poorly defined for the acute and chronic phases of CHIKV infection. Recent findings derived from CHIKV-infected patient cohort studies have identified blood monocytes as cell targets during the viremic phase of acute infection [12]. Not surprisingly, blood monocytes were also shown to play a central role in triggering host innate immunity for other clinically important arboviruses, including Dengue virus (DENV) and Ross River virus (RRV) [13,14].

### 1.1. Epidemiology and Global Expansion

Historical evidence indicates that CHIKV originated from Central/East Africa [15]. Since then, CHIKV has extended its geographical range to various parts of Africa, islands in the Indian Ocean, Europe, Asia, and most recently to the Americas [4,16,17,18]. The 2005 outbreak in La Réunion has been the most severe thus far as it affected up to 34% of its population with 240 deaths [19]. The predominant transmission vector for this wave of outbreaks was the *Ae. albopictus* mosquito, as it preferentially transmitted the CHIKV Eastern/Central/Southern African (ECSA) variant with the A226V amino acid substitution in the E1 envelope glycoprotein [6,7]. Subsequent mutations in the E1 and E2 glycoprotein further adapted CHIKV transmission by the *Ae. albopictus* mosquitos [8]. This adaptation was further demonstrated by the outbreaks in India in 2006 where close to 1.39 million cases were reported [20]. The subsequent outbreaks in Italy in 2007 [21], and in Singapore in 2008 and 2013 further emphasized the ability of CHIKV to thrive within urbanized areas and not just primarily in developing countries [22,23]. Other countries in Southeast Asia were also hit with CHIKV during this period, with outbreaks occurring in Malaysia [24], Thailand [25] and rural villages in the Philippines [26]. The recent CHIKV outbreaks in the Caribbean Islands [18] have raised the possibility of a worldwide transmission. Thus, in the absence of approved vaccines and effective anti-viral remedies, it is of heightened importance that new control strategies are in place.

### 1.2. Challenges in Investigating CHIKV Immunopathophysiology

The development of good CHIKV therapeutics and vaccines remains a challenge due to the inability of the current animal models to adequately recapitulate the immunological response observed in patients [27]. The presence of subtle genetic sequence differences between humans and animal models could result in different phenotypes, or altered individual gene functions [28]. In addition, absence of human-specific genes such as leukocyte defensins and expression of MHC II [29], limit animal models as an efficient tool for predicting human gene function and preclinical drug studies [30]. A “customized” animal model that can provide a closer representation of the human immunological response should be explored in order to give a better illustration of the ongoing mechanisms during CHIKV pathogenesis and disease development.

## 2. Current CHIKV Research on Available Mouse Models

Usage of animals models genetically and taxonomically related to humans has provided an invaluable opportunity to understand CHIKV-associated disease in an *in vivo* setting [31]. The C57BL/6 mouse model is widely used in biomedical research due to its substantial genetic homology with humans [31,32], easy access, and low maintenance cost. Recent innovations in mouse molecular genetics have allowed genomic manipulations of target genes that led to the generation of an impressive range of mutant mice (Table 1). Therefore, the functions of specific genes were explored and assessed to further understand CHIKV infection and immunity.

**Table 1:** Mouse models used in *in vivo* experimental study of CHIKV.

Type of Mice	Mode of Infection	Outcome Indicators	References
A129, AG129 mice	Intraperitoneal (i.p.)	Weight loss, mortality, viremia, histological damages, antibody titer	Partidos *et al.* [33]
WT C57BL/6 mice	Ventral side of footpad (subcutaneous (s.c.))	Footpad inflammation, viremia, histological damages	Gardner *et al.* [34], Morrison *et al.* [35]
Neonatal Swiss albino mice	Intracerebral	Mortality, brain histology	Ross [36], Suckling *et al.* [37]
BALB/c mice	Intranasal infection	Immunohistochemistry	Powers *et al.* [16]
C57BL/6J or NIH Swiss mice	Intranasal infection	Viremia, histological damages	Wang *et al.* [38]
Newborn/neonatal of ICR and CD1 mice	s.c. at the back	Mortality, viral load in organs, histological damage	Ziegler *et al.* [39]
Weaning CD1 mice	s.c. at the rear footpad	*In vivo* bioluminescence imaging of infectious clone	Ziegler *et al.* [40]
CCR2^−/−^ C57BL/6 mice	s.c. at the rear footpad	Footpad inflammation, viremia, viral load of organs, immunohistochemistry, histological damage	Poo *et al.* [41]
Wild-type, IFNAR^−/−^, ISG15^−/−^ and UbE1L^−/−^ C57BL/6 mice	s.c. at right flank	Mortality, cytokine analysis, viral load of organs	Werneke *et al.* [42]
IFN-α/βR^−/−^ and IFN-α/βR^+/−^ outbred OF1 or C57BL/6 mice	Intradermal	Mortality, viral load of organs, histological damage	Couderc *et al.* [43]
IFN-α/βR^−/−^, Cardif^−/−^, RIG-1^−/−^, Mda5^−/−^, Myd88^−/−^, TLR3^−/−^ outbred OF1 or C57BL/6 mice	Intradermal	Mortality, immunohistochemistry, viral load of organs	Schilte *et al.* [44]
CD4^−/−^, μMT, IFNγ^−/−^ C57/BL6 mice	Ventral side of footpad (s.c.)	Viremia, footpad inflammation, antibody response	Lum *et al.* [45]
CD4^−/−^, CD8^−/−^, IFNγ^−/−^ C57/BL6 mice	Ventral side of footpad (s.c.)	Viremia, footpad inflammation, histological damage, *in vivo* imaging	Teo *et al.* [46]
Tlr3^−/−^ C57/BL6 mice	Ventral side of footpad (s.c.)	Viremia, footpad inflammation, histological damage, *in vivo* imaging, immunohistochemistry, cytokine analysis, antibody response	Her *et al.* [47]
IRF3^−/−^, IRF7^−/−^, IRF3/7^−/−^ DKO C57BL/6 mice	Intradermal	Mortality, viremia, viral load of organs, IFN expression analysis	Schilte *et al.* [48]
Rsad2^−/−^ C57BL/6 mice	Ventral side of footpad (s.c)	Viremia, footpad inflammation, histological damage, immunohistochemistry, gene expression analysis	Teng *et al.* [49]
DEREG with IL-2 Ab Cx treatment C57BL/6 mice	Ventral side of footpad	Viremia, footpad inflammation, histological damage, lymphocyte profiling	Lee *et al.* [50]
Neonatal CD1 mice	Intradermal ear injection or mosquito inoculation	Cytokines analysis	Thangamani *et al.* [51]
Newborn Swiss albino mice	s.c. at the back	Proteome analysis	Dhanwani *et al.* [52]

Notes: Newborn mice are defined as 1–3 days old, while neonatal mice are defined as 1–3 weeks old. When not indicated, adult mice were used.

### 2.1. Study of CHIKV Infection and Pathology

#### 2.1.1. C57BL/6 Mice

The C57BL/6 mouse model was first reported as a relevant animal model for CHIKV infection because of its ability to recapitulate several disease manifestations in humans [53]. Variations of mice with the C57BL/6 background, such as neonates and IFNAR^−/−^ adult mice were explored because they lack a fully competent innate immune system, and therefore displayed more severe muscle and joint pathologies [39,42,43,44]. However, the accurate reflection of CHIKV pathophysiology in these models was a concern since they were either immune-deficient or immunologically immature.

Fourteen-day-old C57BL/6 mice were also shown to exhibit CHIKV-induced disease manifestations three weeks after virus inoculation [35]. Histologic analyses of the virus-inoculated hind limb sections revealed severe necrotizing myositis, mixed inflammatory cell arthritis, chronic active tenosynovitis, and multifocal vasculitis [35]. Viral RNA was also detected in musculoskeletal tissues three weeks after infection [35].

#### 2.1.2. Roles of IFN-α/β and Other Immune Mediators during CHIKV Infection

Current findings from CHIKV-elicited immune responses suggest the dual roles played by the host’s innate immune system in regulating viral dissemination and arthritic manifestation. Patient clinical samples showed that viral elimination occurred before the host adaptive immune response was elicited [3]. Substantial amounts of pro-inflammatory cytokines were also observed during the acute phase of CHIKV infection, indicating the involvement of host innate immune cells and mediators in CHIKV disease pathogenesis [54,55,56]. High levels of cytokines detected during the acute phase of CHIKV infection in patients include IFN-α/β, IL-1β, IL-2R, IL-5, IL-6, IL-7, IL-10, IL-12, IL-15, IL-18, GM-CSF, IFN-γ, IP-10, MCP-1, and MIG [54,55,56,57,58]. The contribution of IFN-α/β towards CHIKV clearance was further explored and demonstrated *in vitro* [12,22]. Studies in the macaque model [59] also showed up-regulation of IFN-α/β. The release of IFN-α/β triggers the induction of many anti-viral IFN-stimulated genes (ISGs), including viperin, protein kinase R, 2,5-oligoadenylate synthetase, Mx proteins, ISG15, IRF3, IRF7, and RIG-I, mediating effective CHIKV clearance [42,48,49,60,61]. The importance of IFN-α/β has been well reported in the IFN-α/βR^−/−^ mouse model with higher viral load and severe disease manifestations upon CHIKV infection [62]. In addition, these mice also showed a higher mortality rate [44] when compared to 100% survival in CHIKV-infected wildtype (WT) C57BL/6 mice.

#### 2.1.3. Roles of Adaptive Immune Responses against CHIKV Infection

The adaptive response in C57BL/6 mice does not contribute to the establishment of CHIKV-induced persistent arthritis. However, it has a significant role in the control of affected tissues and virus persistence [63]. CHIKV RNA was detected in a variety of tissues in both WT and Rag1^−/−^ mice very early after infection. CHIKV RNA was still detectable in joint-associated tissues 16 weeks after infection [63]. This phenomenon was observable in both WT and Rag1^−/−^ mice, signifying that the establishment of persistent CHIKV-induced arthritis was not mediated by the adaptive immune system. However, the presence of higher viral load in Rag1^−/−^ mice indicated that the adaptive immune responses control CHIKV infection. The administration of CHIKV-specific monoclonal antibodies prevented the establishment of CHIKV infection, further supporting the protective role of the adaptive immune system [63].

B cells were shown to be critical in the control of CHIKV replication and pathogenesis. There was significantly higher and more persistent viremia in B cell knockout (μMT) C57BL/6 mice [45]. Moreover, μMT mice showed a more pronounced and prolonged joint footpad inflammation compared to WT mice [45].

On the other hand, CD4^+^ T cells were shown to be specifically involved in CHIKV pathogenesis. Mice deficient in CD4^+^ T cells had reduced footpad inflammation and less tissue damage compared to the WT [46]. The same observations were noted when CD4-depleting antibodies were used [46].

### 2.2. Inadequacy of the Current Mouse Models

Although the C57BL/6 model has been widely used to study *in vivo* CHIKV pathogenesis, it has several incongruities when compared to the disease manifestations in humans. These mice lacked signs of polyarthralgia and chronic inflammation, which are typical clinical symptoms observed in patients. The lack of polyarthralgia may be due to the differential modes of virus inoculation between patients and mouse models. The mechanisms involved in the dissemination of CHIKV remain largely unknown, although previous studies on other arboviruses, such as West Nile virus [64] and Cache Valley virus [65] have shown that anti-inflammatory T_H_2 responses elicited by mosquito saliva facilitated viral transmission. Subsequent studies also showed that needle injection of CHIKV induced a pro-inflammatory T_H_1 response and suppressed the anti-inflammatory T_H_2 response, opposite from the immune response elicited by CHIKV inoculation through mosquito bites [51].

Persistent arthralgia has also been reported in approximately 30% of CHIKV-infected patients [9], but no current mouse models are capable of mimicking the chronic disease manifestations. Although adult cynomolgus macaques (*Macaca fascicularis*) have been shown to successfully recapitulate the chronic arthralgia seen in patients [59], it is extremely difficult to work with them due to their high maintenance cost and several ethical issues. Thus, there is a compelling need to identify or engineer a mouse model that is capable of recapitulating the chronic symptoms seen in patients.

## 3. Humanized Mice as Another Alternative Model

The hu-mice humanized mice are mouse-human chimeric mouse models that are quickly gaining popularity. The humanized hematopoietic stem cells (hu-HSC) mouse model and the humanized bone marrow, liver and thymus (hu-BLT) mouse model are the two leading hu-mice models being employed [66]. Each of the aforementioned hu-mice offers a set of unique advantages that can be exploited. The immunodeficient NOD scid gamma (NSG) hu-HSC mouse model supports the highest levels of hematopoietic stem cell engraftment as compared to other humanized mouse models [66,67,68,69]. The hu-BLT NSG mouse model, generated by the transplantation of fetal bone marrow, liver and thymus into NSG mice [70] offers the most functionally complete human immune system amongst the hu-mice models. Moreover, with a transplanted human thymus, T cells are properly educated with an improved adaptive immune system [66]. The hu-BLT mouse model is also the only model offering a functional human mucosal immunity, thus allowing the study of pathogens such as Human Immunodeficiency virus (HIV) [70].

### 3.1. Current Research Using Humanized Mice

Hu-mice models have played an essential role in the study of a wide range of human pathogens, especially those that specifically infect human-blood lineage cells. Significant advancement in the studies of viruses such as HIV Type 1 (HIV-1), Epstein-Barr virus (EBV), and Hepatitis C virus (HCV) were carried out in hu-mice [66].

The hu-BLT mouse model has enabled the study of HIV-1 in a physiological setting [71,72,73]. The model allowed HIV-1 susceptible cells like human CD4^+^ T cells to be differentiated, making it a suitable *in vivo* experimental system [71]. CD4^+^ T cells were demonstrated to have a pathogenic role in CHIKV-induced joint pathology in C57BL/6 mice [46]. Therefore, proper differentiation of human CD4^+^ T cells in the hu-BLT mouse model can be exploited to further define the mechanism of CHIKV pathogenesis.

The inoculation with EBV in NOD-SCID mice reconstituted with human HSC resulted in EBV infection with lymphoproliferative disease. Although the expression of latency proteins indicated type III latency, T cell responses were not adequately established in this model [74]. Improvements made to this mouse model resulted in the development of the hu-HSC RG mice. This model had better human HSC reconstitution and effector T cell generation, which in turn mediated EBV infection and efficient protective immune response against EBV [75]. EBV infection in both hu-HSC and hu-BLT mice exhibited similar results [76]. Given that T cells are important drivers in CHIKV pathology, these models can be explored to provide a better understanding of the functional roles of human T cells in CHIKV pathogenesis.

The lack of human-specific factors that aid in CHIKV entry into susceptible cells, as well as improper stimulation of the host immune system against CHIKV could also be plausible reasons for the limited recapitulation of CHIKV clinical manifestations in the current mouse models. In the HCV disease models, it was demonstrated that despite the expression of viral entry factors such as human Occludin (OCLN) [77] and CD81 [78,79] in transgenic C57BL/6 mice, *in vivo* infection with HCV was restricted [80]. However, HCV infection was successful in the AFC8-hu HSC/Hep humanized mice [80]. Disease manifestations were efficiently recapitulated in this model with HCV inducing liver inflammation, hepatitis, and fibrosis, and eliciting an efficient human T cell response [81]. Thus, it would be of interest to further define specific host factors responsible for disease manifestations in order to further improve the utilization of the current models.

### 3.2. Current Challenges and Limitations of Humanized Mouse Models

Although humanized mouse models have tremendously improved reconstitution of a functional human immune system, the system is not yet ideal [82]. Residual innate immunity in the immune-deficient mouse strains could hamper proper reconstitution of the human immune system. There is also a lack of HLA molecules for appropriate T cell selection in the hu-HSC mouse, and a lack of appropriate HLA antigen presenting cells in the hu-BLT model, resulting in a less than ideal T cell response [82]. The maturation of B cells and antibody production is also less than optimal, and may be attributed to the inefficient cooperation between T and B cells. Native murine cytokines and growth factors have also proven to be species-specific, and are not cross-reactive with human cells [68].

#### 3.2.1. Boosting Cytokines to Improve Immune System Responses

Cytokines are a group of widely classified small proteins essential for intercellular signaling and communication. Significant divergence in genetic sequence exists between several human and mouse cytokines, causing a lack of functional cross-reactivity in the humanized mouse model [67]. The lack of interaction between the cytokines synthesized by mice and the engrafted human HSCs in humanized mice results in deficiencies in species-specific signaling that supports survival, development and function of reconstituted human cells [83,84,85,86]. In order to improve the levels of HSC reconstitution and the overall function of the reconstituted human immune system in humanized mouse, the levels of human cytokines in humanized mice must be boosted. The creation of transgenic NSG mice expressing human stem cell factor (SCF), granulocyte-macrophage colony-stimulating factor (GM-CSF), and interleukin-3 (IL-3) allow limited improvements in human HSC reconstitution upon transplantation of CD34^+^ fetal liver cells. However, a notable increase in the number and function of CD4^+^ FoxP3^+^ human regulatory T cells was also detected [87]. There is little control in the integration of human genes in transgenic mice, which can make it difficult to accurately reproduce the complex spatiotemporal expression pattern of cytokines *in vivo* [87]. To overcome this limitation, specific mouse cytokine genes were substituted with their corresponding human counterparts [88]. The development of multiple knock-in models enhanced reconstitution and function of specific human cells.

#### 3.2.2. Human HLA Transgenic Mice and T Cell Education

Several groups have developed human HLA transgenic mice in an attempt to improve T cell maturation and response towards infection [89,90,91]. The use of HLA-A2 transgenic NSG mice engrafted with HLA-A2 HSC successfully generated HLA-A2 restricted human CD8^+^ T cells upon infection with DENV or EBV [92,93]. As T cells were shown to play a pathogenic role in CHIKV pathogenesis [46], it is important that both T and B cell responses are refined as much as possible to mimic their actual functions. This will result in a better representation of the human adaptive immune response against CHIKV infection. Cognate interactions of T and B cell receptors with immunodominant epitopes have been demonstrated to be critical for mounting an effective immune response against CHIKV. With an improved HLA system in the humanized mouse models, the recognition and presentation of CHIKV antigens in patients will be more accurately represented and further aid in vaccine design and development. However, the current efficiency of the reconstituted T cell response within hu-HSC and hu-BLT mouse models are not up to the mark and improvements would be needed.

## 4. Conclusions

Due to the limitations of the current available non-chimeric mouse models, humanized mouse models can be a plausible alternative for studying CHIKV pathogenesis. The use of humanized mouse models has supported advances in the study of other human viral diseases, and will be beneficial for studying CHIKV pathogenesis. However, the development of humanized mouse models remains exploratory, and additional genetic modifications and manipulations will be needed to significantly improve the *in vivo* recapitulation of CHIKV disease manifestations. Furthermore, the financial cost of generating and maintaining such humanized mice needs to be lowered significantly for long-term sustainability. It is important to note that the humanized mice are man-made engineered animals with inherent flaws. Therefore, these models should act as a supplement to the C57BL/6 models that will continue to be useful in understanding pathogen infection and immunity.
